# Successful cure of multiple large, inoperable liver abscesses by antibiotic therapy

**DOI:** 10.1007/s15010-025-02480-5

**Published:** 2025-02-03

**Authors:** Sarah Niederreiter, Andreas Voelkerer, Christian Datz, Guenter Weiss

**Affiliations:** 1 Department of Internal Medicine II, Medical University of Innsbruck, Anichstr. 35, Innsbruck, A-6020 Austria; 2 Department of Internal Medicine, District Hospital of Oberndorf, Salzburg, Austria

**Keywords:** Inoperable liver abscesses, Antibiotic therapy multiple liver abscesses

## Abstract

**Purpose:**

Pyogenic liver abscesses are challenging due to their diverse etiology and the risk of severe complications. In many cases, surgical interventions are initiated. However, these are only applicable in selected scenarios. We report the case of a 63-year-old woman with multiple large liver abscesses of up to 6.7 cm spread across both liver lobes, which could not be managed surgically.

**Methods & Results:**

*Streptococcus intermedius* was isolated in blood culture and PCR positivity for this pathogen was obtained in liver puncture specimen. Following a two-weeks course of intravenous therapy with cefuroxime, metronidazole and fosfomycin, the patients received a consecutive, combined oral antibiotic treatment with clindamycin and cephalexin for four months. This resulted in complete resolution of the abscesses, with no evidence of relapse at follow-up.

**Conclusion:**

This case illustrates the complex therapeutic challenges in the management of multiple, large hepatic abscesses, highlighting the potential of antibiotic therapy to cure even inoperable patients.

## Introduction

Pyogenic liver abscesses (PLAs) represent a significant clinical challenge due to their varying etiology, complex presentation and the potential for severe complications [1].

The pathogenesis of PLAs often involves ascending biliary duct infections, septic transmission from a focus in the gastro-intestinal tract or in the circulation or invasion per continuitatem. In some cases however, the source of infection remains cryptic [1, 2].

Common causative organisms include the intestinal flora such as *Klebsiella pneumoniae*,* Escherichia coli*, and various Streptococcus/Enterococci species, with *Klebsiella pneumoniae* associated with more severe outcomes and higher rates of metastatic infections [1]. In addition, secondary septic liver abscesses originating from infective endocarditis or other foci in the body often involving also Staphylococci have also to be taken into account. Finally, liver abscesses can also be caused by numerous parasites or helminths including amoeba, fasciola or echninococci [1–3].

The diagnosis typically involves imaging modalities such as ultrasound and computed tomography (CT), which are pivotal in detecting the abscess and guiding subsequent diagnostic and therapeutic procedures [4].

The management of PLAs depends on the presumed causative pathogen and the severity of infection (number, localization and size of abscesses) and involves antimicrobial therapy with either percutaneous drainage or surgical intervention [2, 3]. Specific risk factors have been identified which are associated with a poor outcome of PLAs [5].

Here we describe the case of a 63-year-old female otherwise healthy patient with multiple, large size hepatic abscesses, highlighting the diagnostic complexities and therapeutic decisions involved in treating pyogenic liver abscesses. The purpose of our report is to emphasize that antibiotic therapy can be successful even with very large abscess formation in patients, when surgical interventions are not possible.

### Patient presentation

A 63-year-old female presented to her local hospital due to elevated inflammatory markers and nonspecific abdominal pain. The patient reported a history of diarrhea and fever three weeks ago. Additionally, she had recently undergone dental treatment because of periodontitis. She had no recent travel history, no repeated infections or other co-morbidities. On examination, the patient had diffuse abdominal tenderness in the right lower and upper quadrants as well as mild jaundice of the sclerae. Vital signs were all normal apart from a mild hypotension (RR 98/60 mmHg, body temperature 36,3 C°).

**Fig. 1:**
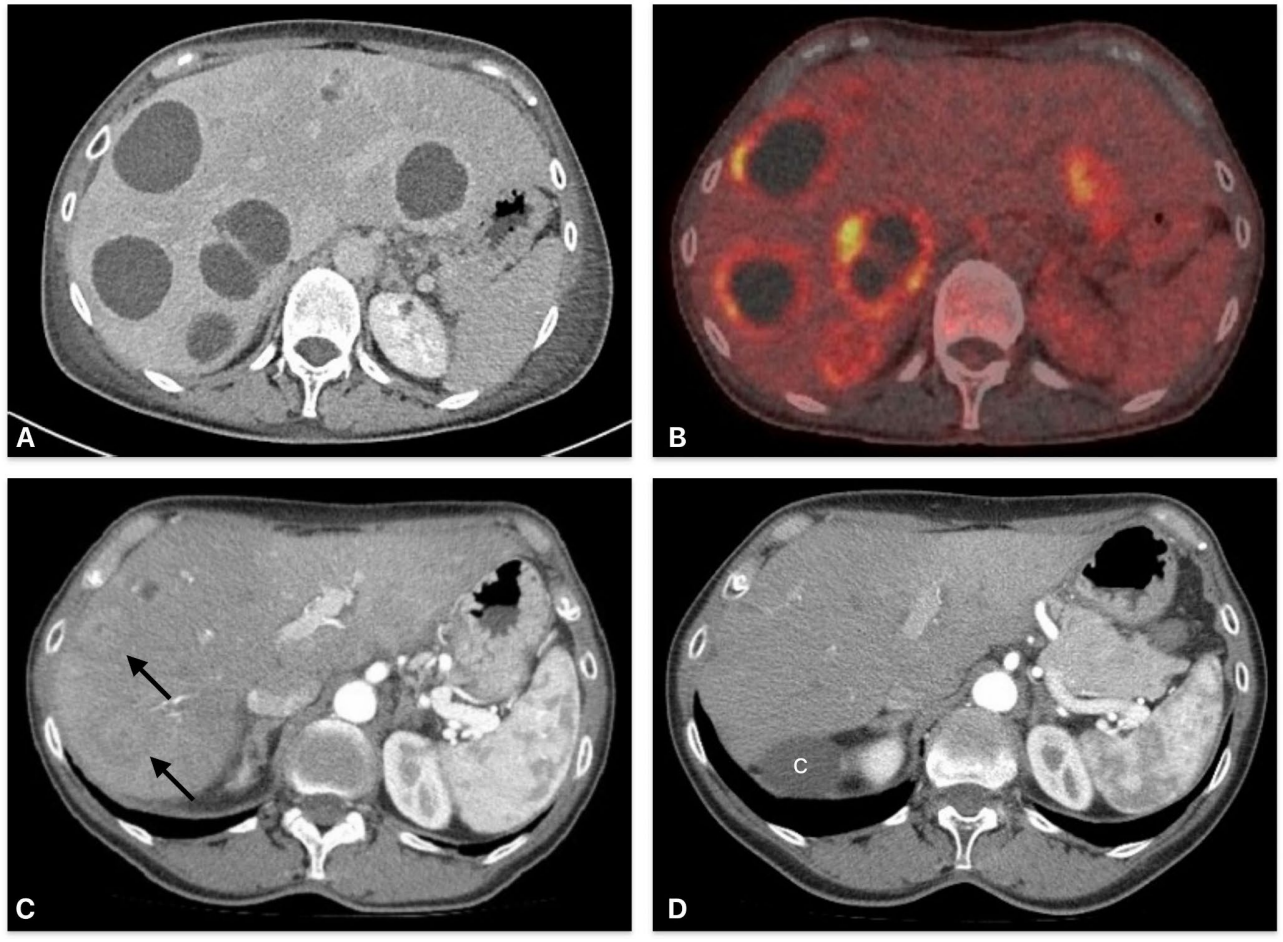
Multimodal imaging of multiple liver abscesses in a 63-year-old female

Initial laboratory results demonstrated massive leukocytosis (31.5 G/L, normal 4.0-10-0 G/L) and elevated C-reactive protein (CRP) levels at 18.32 mg/dL (normal < 0,5 mg/dl). Bilirubin levels were normal with mildly elevated transaminases (AST 71 U/L, ALT 93 U/L, Gamma-GT 195 U/L). An ultrasound of the abdomen revealed multiple hepatic lesions. A parapelvic renal cyst measuring 6.4 cm with peripheral calcifications was also noted. A subsequent CT scan confirmed multiple lesions in both liver lobes, the largest measuring 6.7 cm in diameter. The lesions were smooth and polycyclic, suggesting an inflammatory origin (Fig. 1).

- **A CT scan after initial diagnosis January 29**,** 2020**:
- Multiple abscesses observed throughout the liver, the largest measuring 6.7 cm in diameter
- **B PET-CT results February 10**,** 2020**: Multiple centrally photopenic areas are visualized, which indicate hypometabolic regions consistent with the presence of abscesses
- **C Follow-Up CT scan April 30**,** 2020**: Known multiple liver abscesses,
- Currently shown in axial cross-section with a maximum diameter of up to 4 cm. A slight reduction in size is predominantly observed
- **D Follow-Up CT scan February 17**,** 2021**: Minimal residual findings dorsally
- In the right liver lobe and medially in the left liver lobe, likely representing scarred residues. The other liver abscesses have completely resolved
- Additionally, a previously noted right renal cyst (c) is shown.

Serological tests for echinococcosis were negative twice. Blood cultures identified *Streptococcus intermedius*. Transesophageal echocardiography ruled out endocarditis. Gastro-duodenoscopy and colonoscopy revealed no pathologies. An ultrasound-guided biopsy of a lesion was performed, where purulent material was drained. However, no pathogens were detected because antibiotic therapy has already been initiated meanwhile. Ten days after the initial aspiration, another ultrasound-guided aspiration was performed. Microscopy for pathogen detection and culture results were negative again. *Strepococcus intermedius* was only detected through pan-bacterial PCR of the specimen.

An initial intravenous antibiotic therapy consisted of cefuroxime and fosfomycin, both of which were tested effective against *S. intermedius*. Metronidazol was added to cover anaerobic bacteria. The patient was transferred to our clinical for further evaluation regarding potential surgical intervention. Due to the size and distribution of the abscesses across both liver lobes, surgery was deferred. PET imaging revealed inflammatory activity at the periphery of all abscesses (Fig. 1).

Upon antibiotic therapy including cefuroxime, metronidazole and fosfomycin, signs of inflammation improved significantly over time. The patient was discharged after 15 days and treatment was continued with on an oral antibiotic regimen consisting of clindamycin and cephalexin, both of which tested effective against *S. intermedius*. Follow-up blood cultures remained negative. Oral antibiotic therapy was continued over five months until the patient’s inflammatory markers normalized. Follow-up imaging approximately one year later showed complete resolution of the hepatic abscesses (Fig. 1). The patient had no recurrence of the infection over a follow up period of three years.

## Discussion

Hepatic abscesses are a significant clinical problem due to their potential for severe complications and varying etiology. In this case, a 63-year-old female presented with multiple hepatic abscesses, highlighting the complexity and challenges in managing such conditions.

The diagnostic imaging revealed multiple, heterogeneous hepatic lesions, with laboratory findings showing elevated inflammatory markers and a positive blood culture for *Streptococcus intermedius*. The patients felt well until a few days before the first referral to the local hospital, indicating that hepatic abscesses remain low symptomatic for a long period of time.

The causative pathogen, *Streptococcus intermedius* and part of the Streptococcus anginosus group, is known for causing abscesses in the liver [6].

Infections with *S. intermedius* are exceedingly uncommon in individuals who are otherwise healthy and have no identifiable risk factors. The two most prevalent risk factors are dental procedures and sinusitis [7, 8].

*S. intermedius* can be challenging to identify using conventional microbiological methods, so a broad-range PCR technique targeting the 16 S ribosomal RNA genes is commonly used [9].

While an initial blood culture revealed *S. intermedius*, various cultures obtained directly from the abscess remained negative. The pathogen was detected only through pan-bacterial PCR within the abscess. Pan-bacterial PCR enables the detection of pathogens that can no longer be cultured, whether due to challenging culture conditions or previously initiated antibiotic therapy. Broad-range PCR significantly enhances the detection rate of pathogens even when antibiotic therapy has been started, although it should not replace initial blood cultures taken before the initiation of antibiotic therapy [10].

For pyogenic liver abscesses, CT- or ultrasound-guided percutaneous needle aspiration (with or without catheter drainage) is typically the first-line treatment, achieving success in up to 90% of cases. Catheter placement is recommended for larger abscesses (> 5 cm), though both methods can necessitate repeated procedures. Complications may include organ perforation, pneumothorax, hemorrhage and abscess leakage. If percutaneous drainage is inadequate or in some cases of complex, ruptured, multiple, or percutaneously unreachable abscesses, surgical drainage or laparoscopic drainage may be necessary. Partial hepatectomy is a secondary option due to improvements in percutaneous techniques, while medical management is reserved for high-risk patients or those with small/multiple abscesses not amenable to drainage [11–14]. Of note, it is also important to rule out echinococcosis by serology, if radiological findings are inconclusive, as liver puncture may result in a dissemination of the infection in the peritoneal cavity with systemic infection and sepsis.

In some specific cases, multiple or loculated liver abscesses can be successfully managed by percutaneous drainage, particularly when the abscesses are small and easily accessible. This was illustrated in one retrospective study that described successful percutaneous drainage in the setting of multiple abscesses (22 out of 24 patients) and multiloculated abscesses (51 out of 54 patients) [15].

It is also recommended that in instances with multiple or multiloculated abscesses, the decision between percutaneous drainage and surgery should be made on an individual basis, considering the number, size, and accessibility of the abscesses [15].

While percutaneous drainage and surgical interventions are standard treatments for large and single hepatic abscesses, the decision in this case to avoid surgery was based on the size, the localization and distribution of the abscesses across both liver lobes also targeting the hilus precluding successful surgical intervention. Instead, the patient received a tailored antibiotic therapy regimen, which eventually led to clinical improvement and resolution of the abscesses, as confirmed by follow-up imaging. (Fig. 1)

The selection of antibiotics in this case was guided by several factors including the identification of the causative pathogen, its anti-microbial susceptibility, their potential to penetrate into abscesses in the liver and current clinical guidelines for the management of liver abscesses.

In general empiric antibiotic therapy for liver abscesses should target Enterobacteriaceae, anaerobes, streptococci, and enterococci. Suitable antibiotic regimens include a combination of cephalosporins with metronidazole, beta-lactam/beta-lactamase inhibitors, or in case of multi-resistant pathogens antibiotics penetrating the liver and covering those gram-positive or gram-negative bacteria along with the application of metronidazole [4]. Metronidazole is specifically used to cover anaerobes which are often not detected by anti-microbial testing but are often presented in abscesses. The duration of treatment generally ranges from two to six weeks. After the initial intravenous therapy and clinical improvement and patient’s stability, oral antibiotics can be safely administered in most cases to complete the treatment course [16].

In our case report, the initial antibiotic therapy consisted of cefuroxime and fosfomycin which were both tested effective to *S. intermedius* according to the blood culture result. Cefuroxime is a standard of choice recommended in several guidelines. We added fosfomycin because it has been shown to penetrate well into poorly perfused tissues including abscesses and that it is stable at low pH which is often the case in pyogenic conglomerates [17].

However, due to its high sodium content, fosfomycin may cause hypertension, edema and/or hypokalemia, which have to be carefully monitored. We also added metronidazole according to the guidelines because abscesses are often colonized by anaerobic bacteria which then further contribute to the progression of disease. After successful stabilization of the patient and improvement of the clinical symptoms we switched to cephalexin, which is available at considerably high dosages as oral drug (TID 1 g with excellent oral absorption) and because it penetrates well in tissues and has excellent activity against gram positive bacteria including Streptococci [18].

Clindamycin was used as an empiric combination, because it has a different mode of action against gram positive bacteria by inhibiting bacterial protein synthesis upon to the 50 S ribosomal subunit, thereby preventing peptide chain elongation. Moreover, clindamycin is also well absorbed and offers excellent tissue penetration and effectiveness against abscesses, as it is also effective against anaerobes which have to be suspected in all kinds of abscesses [19, 20]. Notably, both cephalexin and clindamycin were tested to be effective against S. intermedius isolated from the blood culture.

### Limitations

This case report is subject to several limitations: The report discusses a single patient, limiting the generalizability of the findings to broader populations. Variations in clinical presentation and outcomes may occur, making it difficult to draw broad conclusions. The management approach in this case may differ from some standard practices due to individual patient factors, the specific presentation of the disease and clinical needs or contra-indications. This variability can impact the reproducibility of the treatment outcomes in different settings. Without comparative data from similar cases or a control group, it is challenging to generalize the efficacy of the treatment strategy used in this case relative to other potential treatments. Future research and case series can build upon these insights to develop more comprehensive and generalizable conclusions about the management of complicated liver abscesses.

## Conclusion

This case illustrates the complex therapeutic challenges in the management of multiple, large hepatic abscesses, emphasizing the potential of targeted antibiotic therapy to cure even inoperable patients.

## Data Availability

No datasets were generated or analysed during the current study.
